# Towards real-time image deconvolution: application to confocal and STED microscopy

**DOI:** 10.1038/srep02523

**Published:** 2013-08-28

**Authors:** R. Zanella, G. Zanghirati, R. Cavicchioli, L. Zanni, P. Boccacci, M. Bertero, G. Vicidomini

**Affiliations:** 1 Laboratorio delle Tecnologie per Terapie Avanzate, Università di Ferrara, Ferrara, Italy; 2 Dipartimento di Matematica e Informatica, Università di Ferrara, Ferrara, Italy; 3 Dipartimento di Scienze Fisiche, Informatiche e Matematiche, Università di Modena e Reggio Emilia, Modena, Italy; 4 Dipartimento di Informatica, Bioingegneria, Robotica e Ingegneria dei Sistemi, Università di Genova, Genoa, Italy; 5 Nanophysics, Istituto Italiano di Tecnologia, Genoa, Italy

## Abstract

Although deconvolution can improve the quality of any type of microscope, the high computational time required has so far limited its massive spreading. Here we demonstrate the ability of the scaled-gradient-projection (SGP) method to provide accelerated versions of the most used algorithms in microscopy. To achieve further increases in efficiency, we also consider implementations on graphic processing units (GPUs). We test the proposed algorithms both on synthetic and real data of confocal and STED microscopy. Combining the SGP method with the GPU implementation we achieve a speed-up factor from about a factor 25 to 690 (with respect the conventional algorithm). The excellent results obtained on STED microscopy images demonstrate the synergy between super-resolution techniques and image-deconvolution. Further, the real-time processing allows conserving one of the most important property of STED microscopy, i.e the ability to provide fast sub-diffraction resolution recordings.

Image deconvolution is a computational technique that mitigates the distortions created by an optical system. Agard first applied image deconvolution to fluorescence microscopy in the early 1980s^1^. In this seminal paper Agard proposed different algorithms for deconvolving images acquired as three-dimensional (3D) stacks using wide-field microscopy (WFM). In a nutshell, the focal plane of the objective lens moves along the thickness of the specimen and for each position the microscope generates a bi-dimensional (2D) image. Due to the diffraction phenomena, each 2D image, also called optical section, includes considerable out-of-focus light originating from regions of the specimen above and below the focal plane. Image deconvolution uses information describing how the microscope produces the image (forward model) as the basis of a mathematical transformation that reassigns the out-of-focus light to the points of origin.

Later, many new optical methods have been proposed to remove out-of-focus light and to generate directly true optical sections. Without pretending to be exhaustive, we mention confocal laser scanning microscopy (CLSM)^2^,^3^, two-photon excitation microscopy (TPEM)^2^,^4^ and selective plane illumination microscopy (SPIM)^5^,^6^. All these methods remove out-of-focus light by rejecting such light before it reaches the detector or by precluding its generation. Further hybrid techniques, which remove out-of-focus light by combining optical and computational methods are 4Pi microscopy^7^,^8^ and structured illumination microscopy (SIM)^9^,^10^.

Since CLSM, TPEM and SPIM have considerably smaller contribution of out-of-focus light they are sometimes considered as pure alternatives to the deconvolution and WFM combo. However, it has been shown that also these techniques can strongly benefit from image deconvolution^11^,^12^,^13^,^14^. Although out-of-focus background is reduced, the images produced by such systems are still blurred versions of the specimen's structures in the focal plane and are contaminated by noise, thereby deconvolution can improve their contrast and signal-to-noise ratio. Similarly, also single 2D image can benefit of deconvolution, especially when obtained from thin specimen, where out-of-focus background vanishes.

More recently new super-resolution fluorescence microscopy approaches (usually referred to as nanoscopy) have enlarged the portfolio of tools for investigating biological samples^15^. The nanoscopy techniques have effectively break the diffraction barrier and moved the spatial resolution of fluorescence microscopy down to the nanoscale^16^. Importantly, also in these cases image deconvolution can help to improve the quality of their images. This has been demonstrated both for stimulated emission depletion (STED) microscopy^17^, which at moment can be considered as the method of choice between the targeted nanoscopy techniques, and, more recently, also for stochastic nanoscopy techniques^18^. As a matter of fact, all microscopy techniques that include directly or indirectly a convolution in their image formation processes can benefit from image deconvolution. It is also important to remember that any quantitative analysis on fluorescence images, e.g. colocalization analysis or volume/area estimations, are significantly improved if performed on deconvolved images^19^,^20^.

In this scenario, one expects that any 2D or 3D image obtained from almost any fluorescence microscope is deconvolved before being analyzed. This unfortunately is not true. The main disadvantage that precludes this massive spreading of deconvolution is the high computational demand which leads to long waiting time before producing the result. As a consequence in many applications image deconvolution is not used to avoid strong delay in the data analysis pipeline. The situation becomes almost prohibitive in the case of large-scale images. For the above mentioned reasons, several methods to increase the speed of the deconvolution process have been proposed.

Two main directions have been followed. The first one relays on the implementation of the algorithms, *i.e.*, parallelization of the calculus and/or implementation on graphics processing units (GPUs)^21^,^22^,^23^,^24^. A second approach, which found a strong attraction in the 90s, relays on the development of schemes to accelerate the deconvolution algorithms^25^,^26^. Even if linear deconvolution, *e.g.*, Wiener filtering, is extremely fast, its application to noisy images provides in general poor results; on the other side non-linear deconvolution methods, and in particular iterative methods (with or without regularization), lead to excellent results but their convergence is very slow, requiring hundreds or thousands of iterations. The major representative algorithms for non-linear deconvolution in fluorescence microscopy are based on the maximum-likelihood (ML) approach and, for the regularized version, on the maximum *a posteriori* (MAP) approach^27^. These algorithms can take advantage from prior information about the image formation process and the specimen, effectively reducing the ill-posedness of the problem. Most of this algorithms are iterative first-order methods, hence their implementation is easy (basically computation of a matrix-vector multiplication at each iteration), but, as already mentioned above, their convergence is very slow. In this paper we present a deconvolution package that combines both strategies.

Recently, Bonettini et al.^28^ developed an optimization method, which they called scaled-gradient-projection (SGP) method, able to fundamentally speed-up the algorithms based on ML and MAP. In this paper we use the SGP method to derive a more efficient version of the Richardson-Lucy (RL) algorithm^29^,^30^, which represents the most famous non-regularized algorithm for deconvolution on microscopy images. Moreover, the SGP method is also used to derive an acceleration for another important widely used regularized deconvolution algorithm based on a quadratic regularization term^31^. Finally, both algorithms can be integrated by a boundary effect correction according to the approach proposed by Bertero and Boccacci^32^. This correction allows the application of these algorithms also to images of cropped structures.

We have first implemented the algorithms for classical central-processing-unit (CPU)-based calculation, in order to quantify the effective speed-up obtained by the proposed SGP method with respect to RL. Later, we implemented the algorithm for GPU-based calculation to further reduce the time of the deconvolution process. Importantly, codes for the CPU-based implementation will be freely distributed, as well as the executable files for the GPU-based implementation.

The purpose of this paper is not only to illustrate the features of the SGP method to the microscopy community but also to provide them quasi real-time deconvolution algorithms able to drastically reduce the time for the pipe-line image analysis. We used both CLSM and STED microscopy images to demonstrate the speed-up of the SGP based algorithms. However, the very same algorithms can be applied to any other fluorescence microscopy technique by simply providing the relative point-spread-function, or more generally the relative forward model.

## Results

Maximum-likelihood and maximum *a-posteriori* approaches reduce the deconvolution problem into the minimization of a suitable functional (equations (2) and (3)). This functional includes most of the information about the image formation process and, when possible, information about the object to restore, thereby its design represents an important step for the quality of the deconvolution results. On the other hand, the speed of a deconvolution algorithm strictly depends on the scheme used to minimize the functional. Since in this work we focused our attention mainly on the speed issue, we compared the performance of the algorithms when they minimize the very same functionals.

**Figure 1:**
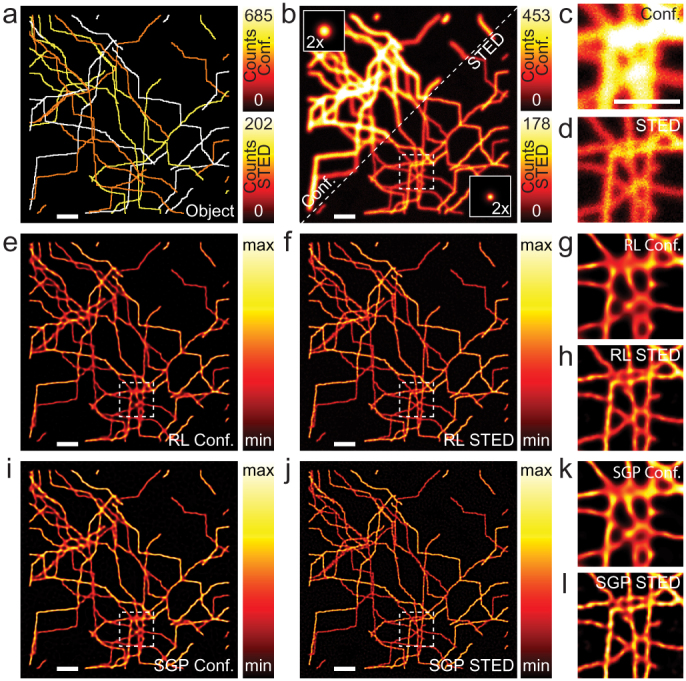
Comparison between SGP- and RL-based restorations of 2D synthetic data. (a) Phantom mimicking the micro-tubulin cytoskeleton of a cell (512 × 512 pixels, 20 nm pixel size). In particular the map depicts the distribution of fluorescent photons being emitted by each pixel. The two color bars represent respectively the CLSM and STED microscopy case. Since STED microscopy improves the resolution by reducing the effective area from which fluorescence is emitted, the amount of photons in the STED microscopy case reduces. (b) Synthetic images for the CLSM (upper left, SNR ~13 db) and STED microscope (lower right, SNR ~11 db). Insets represent the respective PSFs magnified by a factor 2. (c,d) Magnified views of the area denoted by the dashed box in (b) for CLSM and STED microscopy, respectively. (e,f,i,j) RL-based (e,f) and SGP-based (i,j) restorations of CLSM (e,i) and STED (f,j) images. (g,h,k,l) Magnified views of the area denoted by the white box in (e,f,i,j). All the magnified views are renormalized in signal intensity. Algorithms were stopped using the minimization of the KL-divergence as criterium (Eq. (S28)). Scale bars: 1 *μ*m.

We first used bi-dimensional (2D) CLSM and STED microscopy synthetic images for comparing the well-known RL algorithm with the SGP-based algorithm (both minimizing the functional described in equation (2)). Realistic phantoms are crucial when robustness of algorithms has to be evaluated. For this reason, we implemented a routine able to generate pseudo-randomly phantoms which mimic the micro-tubule cytoskeleton of a cell (see Methods). We simulated the images of the two microscopy modalities by using the same random microtubule network specimen (Fig. 1a) and the same imaging conditions (see Methods), but two different point-spread-functions (PSFs) (Insets Fig. 1b) for mimicking the different spatial resolutions. In particular, we assumed a Gaussian shaped PSF (see Methods) with a full-width at half-maximum (FWHM) of 220 nm for CLSM (*σ_r_* = 93 nm) and a Gaussian-Lorentzian shaped PSF (see Methods) with a FWHM of 100 nm for STED microscopy (*σ_r_* = 93 nm, *ψ* = 3.22·10^−3^ nm^−1^, *ς* = 7). Figure 1e–l shows a side-by-side comparison of RL- and SGP-based restorations. Clearly, the STED microscopy image reveals superior details compared to the CLSM image because their differences in spatial resolution (Fig. 1b,c,d, Fig. S1). Importantly, we deconvolved the synthetic images by means of the very same PSFs used for their generation (inverse crime). Thereby the results were not fundamentally biased by the choice of the PSF. After deconvolution we obtained excellent contrast improvement and noise reduction that help distinguishing more structural details into the CLSM restored images (Fig. 1e,g,i,k), as well as, into the STED microscopy restored images (Fig. 1f,h,j,l).

More interesting for the scope of this work is the comparison between RL- and SGP-based restorations. Both algorithms led to similar results (Fig. 1e,f,i,j). However close looks to the restorations (Fig. 1h,l,g,k) depict slight differences. For example the SGP-based images offers higher contrast with respect to the RL-based counterpart. A quantitatively analysis confirmed such improvement (Fig. S1). Even if RL and SGP algorithms converge to the same minimum, they follow different approximation paths and the restorations satisfying the stopping rule can present marginal differences.

**Figure 2:**
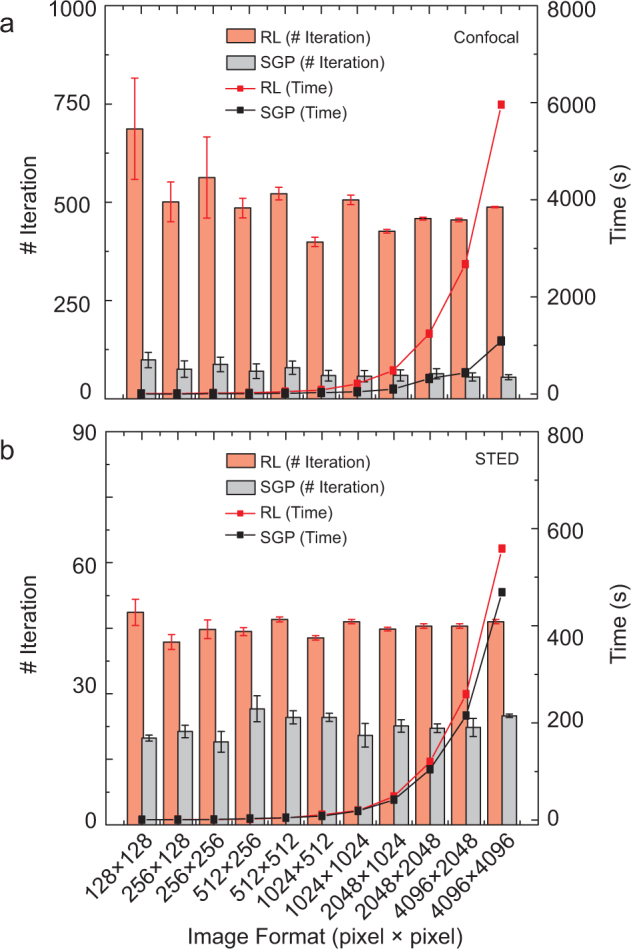
Performance comparison between the SGP and RL algorithms. (a,b) Number of iterations and computational time as a function of image size for CLSM (a) and STED microscopy (b). All the test compared the CPU-based implementations. Each point represents the mean and the standard deviation of 20 different realizations, in particular 10 different noise realizations for 2 different phantom realizations. Same conditions of Figure 1.

Whereas RL and SGP algorithms are similar in terms of restoration quality they have strong differences in terms of speed. Figure 2 plots the time and the number of iterations requested for obtaining optimal (in terms of restoration accuracy (see Methods)) restored images as function of the image size (number of pixels). We confirmed robustness of the algorithms against noise and object structures by running the algorithms with different noise realizations and different random tubulin network realizations. Moreover, we carefully maintained the concentration of filaments constant for all image sizes, in order to remove any dependency of the iterations' optimal number on the image size. On the other side, the optimal number of iteration changes between the two microscopy techniques. However, the reason is not connected to the microscopy technique itself, but to the different intensity-dynamic of their images (Color bars Fig. 1a) and the different size of the their PSFs. As a rule of thumb the optimal number of iterations increases for increasing intensity-dynamic (number of photons collected *per* pixel) and blurring of the image (size of the PSF with respect to the pixel size).

More interesting for SGP and RL algorithms comparison is that SGP reduces the optimal number of iterations (~87% for CLSM and ~51% for STED microscopy). This is in agreement with the main feature of the SGP method, *i.e.*, the ability to find optimal direction toward the minimum of the functional and thereby to reduce the number of iterations needed. However, a fair comparison of the speed-up of SGP algorithm has to take into account that a single SGP iteration needs more computation than a single RL iteration. Thereby, the overall time speed-up obtained with the SGP algorithm is ~20% for STED microscopy and ~80% for CLSM. Similarly to the RL algorithm, also the SGP algorithm decreases the optimal number of iterations when the signal-to-noise ratio (SNR) decreases. Thus, in a regime of very low SNR the speed-up of the SGP-based algorithm with respect to the RL algorithm can reduce (Fig. S2).

**Figure 3:**
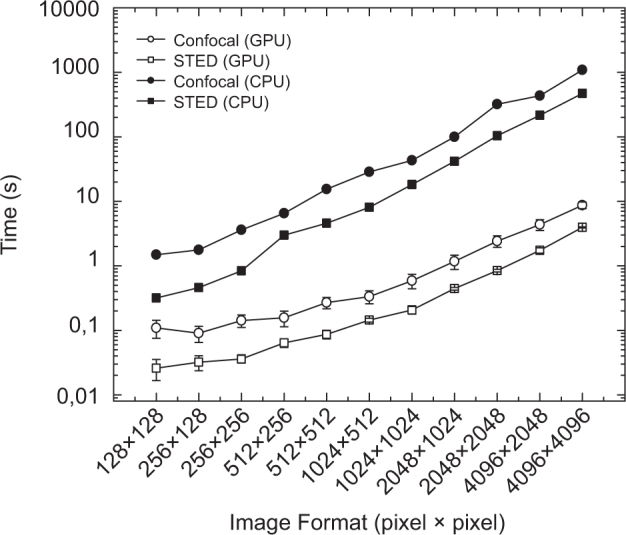
Performance comparison between CPU- and GPU-based implementation of the SGP algorithm. Computational time as a function of image size. Same conditions of Figures 1 and 2.

After estimating the speed-up related to the SGP algorithm alone, we evaluated the further speed-up obtained by implementing the SGP algorithm for GPU (instead of CPU). Figure 3 shows the time needed to obtain optimal restoration as a function of the image size. The GPU-based algorithm works ~10 times faster for small images (126 × 126 pixels) and ~100 times faster for large images (4096 × 4096 pixels) when compared to the CPU-based algorithm. Notably, this speed-up has to be added to the speed-up provided by the SGP algorithm, for example for large CLSM images (4096 × 4096 pixels) the GPU-based SGP algorithm need ~8 s, that is ~690 times faster then the CPU-based RL algorithm. If we consider that to achieve adequate SNR a modern CLSM need a pixel-dwell time of at least 1 *μ*s, in this example the deconvolution process is at least 2 times faster then the time to collect the image.

**Figure 4:**
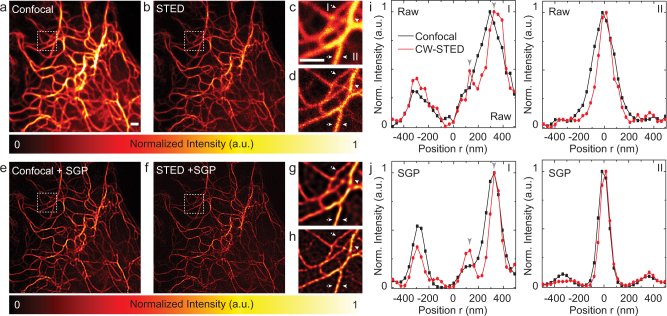
SGP-based restoration of 2D real CLSM and STED microscopy images. (a,b) Bidimensional CLSM (a) and STED microscopy (b) images of the micro-tubulin network of a PtK2 cell (512 × 512 pixel, pixel dwell-time 10 *μ*s, pixel size 32 nm). (c,d) Magnified views of the area denoted by the dashed box in (a) and (b), respectively. (e,f) SGP-based restored image of (a) and (f), respectively. (g,h) Magnified views of the area denoted by the white box in (e) and (f), respectively. (i,j) Intensity profiles (along the lines between the arrows) through single isolated filament (I) or close-packed filaments (II) in the raw (c,d) and restored (g,h) images. Scale bars: 1 *μ*m.

Next, motivated by the promising results on synthetic images we applied the SGP algorithm to real images of tubulin network (Fig. 4). In contrast to results on synthetic images, results on real images strictly depend on the PSF. Thereby, even if any method which estimate the PSF is fully compatible with the proposed algorithms, one has to pay particular attention to the PSF choice. A PSF may be empirical, *i.e.*, measured^33^ or theoretical, *i.e.*, calculated^34^. Empirical PSF is generally obtained by imaging of sub-resolved structures in the same system conditions (i.e. optics and specimen's environment) used to image the specimen. Whereas calculated PSF is generated by using analytical models which require parameters like wavelength configurations, objective lens details, refractive indexes of immersion and mounting media, etc. Both methods present advantages and disadvantages. Briefly, an empirical PSF is contaminated by noise and has to be measured exactly in the same conditions that will be used to image the specimen, on the other side, a measured PSF takes into account any kind of aberration that can arise in the whole system, including aberration introduced by the specimen itself; a theoretical PSF is noise-free, but, its computation requires many information that are not easy to known and complex models. Also, a third option exists where the PSF is estimated from the image together with the unknown object, *i.e.*, blind deconvolution^35^. In this paper we adopted an hybrid method (see Methods): we used a rather easy PSF parametric model whose parameters are directly extracted from the image of sub-resolved structures contained in the very same specimen (*σ_r_* = 93 nm, *ψ* = 3.22·10^−3^ nm^−1^, *ς* = 5.2). Importantly, in the case of deconvolution for STED microscopy is extremely important to estimate the PSF directly from the image being deconvolved since the PSF strictly depends also by the properties of the fluorescent marker. For example, the use of fluorescent beads can result in a wrong estimation of the PSF, since in most of the case the fluorescent marker used for the beads is different from the one used for labeling the specimen.

**Figure 5:**
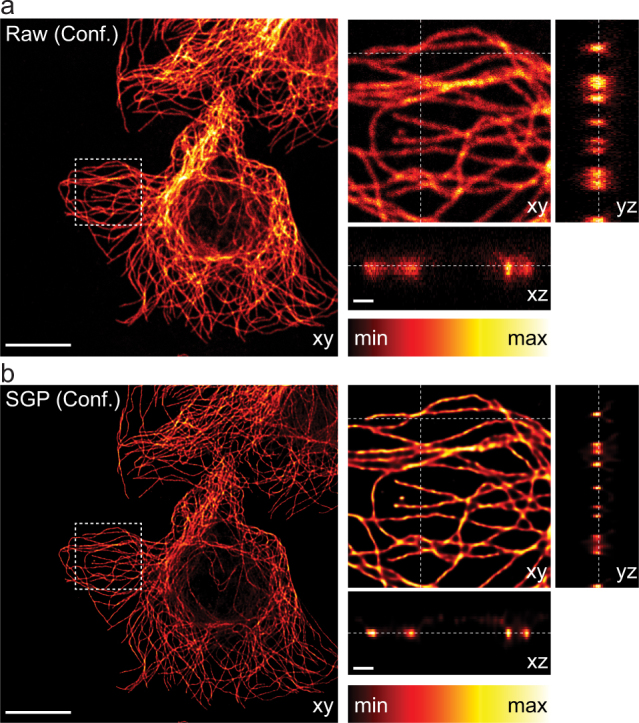
SGP-based restoration of 3D large real CLSM data set. (a) Middle xy section of the raw data (1024 × 1024 × 33 pixel, pixel dwell-time 5 *μ*s, pixel size 50 × 50 × 125 nm). (b) Middle section of the SGP-based restored data (PSF parameters *σ_r_* = 106 nm, *σ_z_* = 297 nm). Insets show the magnified cross-sectional views of the volume denoted by the white box. The dotted lines indicate the position of the xy-, xz- and yz-slices shown for each of the 3D stacks. Scale bars: 10 *μ*m and 1 *μ*m (insets).

The superior resolution of STED microscopy clearly highlights filaments intersection that can not be resolved in the CLSM counterpart (Fig. 4a–d). By strongly improving the contrast and reducing the noise, the SGP algorithm is able to recover many structural details from the raw CLSM image, as well as from the raw STED microscopy images. These results fully confirm the importance of applying deconvolution also to super-resolution techniques, such as STED microscopy. Moreover, this example clarifies which are the benefits of using algorithms based on equation (2), like RL and SGP. It is well known that minimization of equation (2) leads to pointwise (sparse) restorations. For this reason many regularization methods have been proposed by different groups in order to apply deconvolution also for imaging of piecewise structures. In this work we applied deconvolution on tubulin network images, which is a rather sparse structure. SGP algorithm offers superior results when it is applied to reconstruct single isolated tubulin filaments. There are almost no differences between CLSM and STED microscopy restoration when comparing the intensity profile through a single isolated filaments (Fig. 4j), *i.e.*, deconvolution on CLSM can, in these particular circumstances, substitutes STED microscopy. On the contrary, when more convoluted structures are imaged, the lower resolution offered by CLSM microscopy can not be compensated by deconvolution. STED microscopy, especially when combined with deconvolution, easily resolves two close (<100 nm) tubulin filaments (Fig. 4i), but CLSM, even if combined with deconvolution, fails on the same task (Fig. 4i). The GPU-based SGP algorithm provided the restoration in ~0.07 s (21 iterations) and ~0.16 s (45 iterations) for CLSM and STED microscopy, respectively. Indeed, ~37 (STED) and ~16 (CLSM) time faster than the time that the microscope need to produce the images. The advantages of using the GPU-based algorithm becomes plain for 3D data set. We tested the SGP algorithm on a 3D CLSM image of the entire cytoskeleton of a cell (Fig. 5a) which took 180 s to be collected. Despite the huge data set (1024 × 1024 × 33 voxel) the GPU-based implementation of the SGP algorithm obtained an excellent restoration (Fig. 5b) after 20 iterations taking ~35 s, which is about a factor ~5 and ~35 faster than the collection time and the time need by the CPU-based implementation, respectively. Finally, we remark that we obtained all the results working with double precision, thereby a further reduction of running time is expected when using single precision. For example, when working in single precision the entire cytoskeleton 3D restoration needed ~17 s, thereby ~2 time faster. Importantly, we observe that in the microscopy contest running the deconvolution algorithms in single and double precision we obtained similar qualitative results.

## Discussion

Image deconvolution can potentially improve image quality for any fluorescence microscopy technique, including the new emerging nanoscopy techniques. However, the amount of computational time required, which characterizes any high performance algorithm, has so far limited the massive spreading of image deconvolution. In this paper we describe a framework able to efficiently reduced the computational time for solving both the ML (un-regularized) and the MAP (regularized) deconvolution problem. This framework uses the SGP method for solving the minimization problem associated to deconvolution. As an example, we use this framework to derive an efficient alternative to one of the most used deconvolution algorithm in fluorescence microscopy, the RL algorithm. Further, we compared CPU-based and GPU-based implementations of this algorithm. The synergy between the SGP method and the GPU-based implementation achieves an improvement which ranges from about a factor of 25 to 690 (when compared to a CPU-based implementation of the RL algorithm), without loosing in quality of the reconstruction.

The executable files for the GPU-based implementation can be freely downloaded (http://www.unife.it/prisma), as well as the codes for the CPU-based implementation. Moreover, as an example of the SGP method applied to regularized deconvolution, the software provides a GPU-based algorithm which can efficiently substitute the widely used regularized algorithms based on Tikhonov regularization. Last but not least, the software includes a boundary effect correction, which allows the application of the algorithms to images of cropped structures.

It is important however to point out the limitations of the SGP method which, in this paper, is mainly applied to the ML problem because we are focusing on possible real-time applications. As previously remarked, the SGP method can also be applied to the solution of regularized problems (and one example is provided in this paper), but only if the regularization function is differentiable. This is an important limitation because, in general, the SGP method can not be applied to the important case of sparse reconstruction schemes, *i.e.* 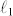-norm regularization. More precisely, it can be applied to the case of edge-preserving regularization if a smoothed TV-norm is used^36^, but not to the case of sparsity of the object with respect to a suitable wavelet transform, such as a dual-tree complex wavelet transform or a dictionary composed of curvelets and un-decimated wavelet transform^37^,^38^, an approach already proposed for confocal microscopy.

In the case of a piece-wise object with sharp edges, regularization by early stopping of un-regularized SGP or RL can produce a smoothing of the edges and therefore edge-preserving regularization is required. This over-smoothing effect does not appear in the restoration of tubulines networks because, as we already remarked, this is essentially a sparse object and the ML solutions are sparse in the pixel space.

In the case of regularized methods an important point is the choice of the regularization parameter. For any selection criterion the solution of several minimization problems is in general required so that a real-time application is not possible. A way could be the approach proposed in^38^, where the choice of the parameter is reduced to the solution of a unique constrained minimization problem with an additional constraint related to the selection criterion. We believe that also this constrained minimization problem is too much time-consuming to enable real-time deconvolution with the available GPU technology. A more practical way could be to calibrate off-line the regularization parameter for a given class of objects (for instance tubulines) and a given value of the signal-to-noise ratio. Then the estimated value could be used for real-time SGP-based deconvolution.

We conclude by highlighting the advantages of image deconvolution on STED microscopy imaging. The question if conventional microscopy combined with image deconvolution alone (without using prior information about the object to reconstruct) can recover object's frequencies beyond the cut-off frequency of the system (*i.e.* achieve sub-diffraction resolution) is still controversial. In the case of STED microscopy the situation is rather different. In a STED microscope the response of the object's emission rate to the illumination is nonlinear (exponential). Roughly speaking, this property allows to the STED microscope system to transfer all the object's frequencies (no cut-off frequency exists), thus permitting theoretically unlimited resolution^39^,^40^. However, the strength of the frequencies declines rapidly with the increases of the order, leaving the practical resolution finite due to signal-to-noise concerns. On the other side, in a STED microscope the strength of the high frequency can be enhanced by increasing the intensity of the illumination, which unfortunately can also introduce photodamage effects on the specimen. In this scenario, image deconvolution can efficiently help recovering high frequencies which are transmitted by the microscope system but hindered by the noise, thereby improving the practical resolution without increasing the intensity of the illumination.

## Methods

### Scaled-gradient-projection (SGP) algorithm

Let us assume that the image detected values *y_i_* (here *i* is an index labeling the pixels or voxels of the image) are realizations of independent Poisson random variables, with unknown expected values (*Hx* + *b*)*_i_*, where *x* is the unknown object, *H* is the imaging matrix given in terms of the known PSF of the microscope *h* by 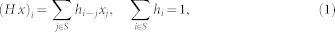
and *b* is the known background emission. Then, the maximum-likelihood (ML) approach to the image deconvolution problem is equivalent to minimize the following generalized Kullback-Leibler (KL) divergence (or Csiszár I-divergence)^27^, given by 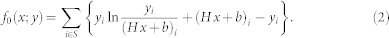
As shown in^41^, an iterative algorithm converging to nonnegative minimizers of the KL divergence is the well-known Richardson-Lucy (RL) algorithm^29^,^30^, recalled in the Supplementary Information.

Since it is known that the nonnegative minimizers of the generalized KL divergence consist of a set of bright spots over a black background, the so-called night-sky solutions^42^, the algorithm can not be pushed to convergence and early stopping of the iterations is required for obtaining a sort of “regularization” effect. Recently a few stopping criteria have been proposed^43^,^44^,^38^, but their utility in practice has still to be tested.

Regularization can also be obtained in a Bayesian framework by assuming that the unknown object *x* is a realization of a random variable. If the probability density (prior) is of the Gibbs type, by taking the negative logarithm of the posterior probability one finds that the maximum *a-posteriori* (MAP) estimates are the nonnegative minimizers of the function 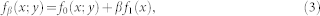
where the second term is the negative log of the prior. In the following we will call *f*_1_(*x*) the regularization function and *β* the regularization parameter. Examples of *f*_1_(*x*) considered in microscopy are, for instance, the square of the 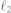 norm of *x*^31^ or edge-preserving functions of *x*^45^,^46^.

In the case of a differentiable penalty function *f*_1_(*x*) several iterative methods have been proposed for the minimization of the function *f_β_*(*x*; *y*) defined in equations (3) and (2). For our purposes two methods are interesting: the one-step late (OSL) method proposed in^47^ and the split-gradient method (SGM) proposed in^48^. The first is used for instance in^45^ for total variation (TV) regularization and the second in^46^ for Markov random field (MRF) regularization.

It is easy to show that both OSL and SGM are scaled gradient methods; however only in the case of SGM the scaling is nonnegative for any regularization function *f*_1_(*x*) and any value of the regularization parameter *β* (see Supplementary Information). Therefore our reference algorithms are RL for the maximum-likelihood approach and SGM for the Bayes approach.

Motivated by the large application of these algorithms in microscopy, we derived new algorithms based on the scaled gradient projection (SGP) method^28^ which use the scaling suggested by the RL (Eq. (S3)) and the SGM (Eq. (S6)) algorithms. In the first case SGP is able to provide a very efficient solution of the ML image deconvolution, hence an acceleration of the RL method; in the second case an efficient solution of the MAP image deconvolution with 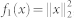, hence an acceleration of the algorithm proposed in^31^. For the purpose of this paper, we describe the SGP algorithm in the case of diagonal positive definite scaling matrices and nonnegativity constraint. The general case of arbitrary convex constraints and/or non-diagonal positive definite scaling matrices is given in^28^.

The considered ML and MAP problems are particular cases of the following general convex optimization problem 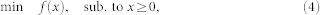
where *f* is a continuously differentiable convex function. The SGP algorithm for solving this problem can be stated as in Table 1.

Here we denote by 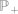 the projection onto the nonnegative orthant, *i.e.*, the operator setting to zero the negative components of a vector, and by 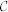 the compact set of the diagonal positive definite matrices whose diagonal entries have values between two positive constants *L*_1_ and *L*_2_, 0 < *L*_1_ < *L*_2_.

As a gradient projection algorithm, SGP involves two standard elements: the choice of a descent direction (Step 3.) by means of the projection onto the feasible region and a line-search along the descent direction (Step 5.). For the latter, a classical monotone line-search technique is considered but, as described in^28^, nonmonotone strategies could be also exploited. The main feature of SGP consists in the definition of the search direction, that is obtained by combining diagonally scaled gradient directions with special step-length selection rules with the aim of accelerating the path toward the minimum without losing the simplicity and low computational cost of each iteration. In particular, the choice of the step-length *α_k_* is usually inspired by quasi-Newton properties, but without the need of computing any second-order information. In our implementations we use an adaptive alternation strategy based on the two Barzilai-Borwein (BB) rules which, in the case of a scaled gradient directions, are as follows^28^

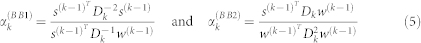
where *s*^(*k*−1)^ = *x*^(*k*)^ − *x*^(*k*−1)^ and *w*^(*k*−1)^ = ∇ *f*(*x*^(*k*)^) − ∇ *f*(*x*^(*k*−1)^). When *D_k_* is equal to the identity matrix, one obtains the standard BB rules^49^. More details on the adaptive step-length alternation rule used in SGP are given in the Supplementary Information and we refer to^50^,^51^,^52^ for discussion on the rationale behind the step-length alternation. Concerning the choice of the scaling matrix *D_k_*, it takes into account the special form of the function *f*(*x*) we are minimizing and needs to be faced separately for each application considered in the paper. In the case of the minimization of the KL divergence we use the scaling suggested by Equation (S3), corrected with a threshold assuring that the scaling matrix belongs to 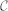


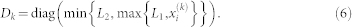
Similarly, the analysis of SGM suggests the following scaling in the application of SGP to the minimization of *f_β_*(*x*; *y*) 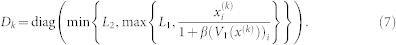
where *V*_1_(*x*^(*k*)^) is a nonnegative array/cube defined by an appropriate splitting of ∇*f*_1_(*x*^(*k*)^). In the case of quadratic (or Tikhonov) regularization, we recall that *V*_1_(*x*^(*k*)^) = *x*^(*k*)^ (see Supplementary Information). Here it is also shown that, as far as the SGP algorithm concerns, the boundary effect correction is incorporated in the scaling matrix while all the other steps remain unchanged.

We conclude by recalling that global convergence of the algorithm is proved in^28^, for every choice of the step-length *α_k_* in the closed interval [*α*_min_, *α*_max_] and of the scaling matrix *D_k_* in the compact set 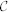. Further useful implementation suggestions on the variables initialization, the parameter setting and the stopping rules can be found in the Supplementary Information.

### Point spread function

It has been shown by^53^ that a confocal PSF is well modeled by a radially symmetric Gaussian function as: 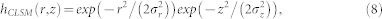
where *σ* is related to the full-width at half-maximum (FWHM) by 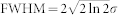. We estimated both *σ_r_* and *σ_z_* from the detected confocal image by using intensity profiles of sub-resolved structures into the image, like unspecifically bound single antibodies or nanosize sub-cellular compartments, together with Gaussian fits for obtaining *σ_r_* and *σ_z_*. Similarly, it has been shown that the PSF of a STED microscope operating with continuous-wave (CW) lasers (also called CW-STED microscope) is well modeled by^54^,^55^: 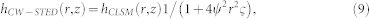
where: ψ is a constant that depends on the shape of the doughnut-like STED intensity distribution at the focus^56^; *ς* is the so called saturation factor, which is defined as *ς* = *I_STED_*/*I_s_*, *I_STED_* being the maximum value of the STED intensity distribution at the focus and *I_s_* being the effective saturation intensity, which can be defined as the intensity at which the probability of fluorescence emission is reduced by half. In the most general case, *I_s_* is a function of the orientation distribution and rotational behavior of the fluorescent marker, as well as of the wavelength, temporal structure and polarization of the inhibition light^56^,^57^. We estimated ψ by using scattering images of single isolated 80-nm gold bead. Importantly, *ς* = 0 and *h_CW_*_−*STED*_ = *h_CLSM_* if the STED beam intensity is null. Thereby, by taking advantage of having the CW-STED and confocal images of the very same specimen, we estimated the *σ* values as described above. Next, given *σ* and ψ, the saturation factor *ς* was estimated using equation (9) and the intensity profiles through sub-resolved structures in the CW-STED image. Finally, we mention that the Gaussian-based models for the PSF can fail in the case of thick specimen. In this case images are affected by a depth-variant blur due to spherical aberrations induced by refractive index mismatch between the different media composing the system as well as the specimen^58^. Nevertheless, in practice it is difficult to obtain such a PSF, in spite of the existence of theoretical models accounting for spherical aberrations, because these models depend on some unknown acquisition parameter, such as the refractive index of the specimen. Therefore one needs blind or semi-blind restoration algorithms (see, for instance^59^, where an alternating minimization scheme is used in conjunction with SGP as minimization algorithm for depth-variant image deconvolution in confocal microscopy).

### Synthetic images

To generate pseudo-random phantoms which mimic the micro-tubule network of a cell we randomly selected the starting positions of a given and fixed number of filaments and successively we used a stochastic process for choosing iteratively the directions of growth. The growth has been performed in a bi-dimensional or three-dimensional space to obtain 2D or 3D phantom, respectively. We assumed filaments having tubular structure with radius 30 nm and we introduced heterogeneity of protein concentration between different filaments by associating to each filament a value in the range [0, 1]. Successively, to obtain the ideal image we convolved the phantom 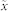 with the system PSF, *i.e.*, 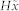. Importantly, the PSF of the STED system becomes narrower respect to the confocal counterpart as the saturation factor *ς* increases, but the intensity value at the peak stays constant. Thereby, in the convolution process, we used Equations (8) and (9) without any normalization to the sum of the pixels/voxels.

To obtain the ideal image in terms of average detected photons, we multiplied the convolved object by a factor *τ* which depends on several multiplicative factors, such as the emission rate of the fluorophore, the collection efficiency of the system and the pixel dwell-time. Since we assumed that photon counting noise represents the major source of noise for the detection process, and a constant background *b* can further degrade the image, we obtained the final image by corrupting every pixel/voxel *i* with a Poisson process with mean 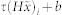. Thus by increasing *τ*, the average number of detected photons increases and hence the signal-to-noise ratio (SNR) increases. The relation between SNR and *τ* is 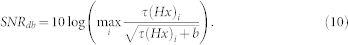
Since in simulation we know the object 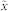 used to generated the simulated image, we can numerically evaluate the quality of the deconvolved images at each iteration *k*. In particular, we used the Kullback-Leibler divergence of the reconstructed image *x*^(*k*)^ from the known object 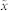 (see Supplementary Information). Notably, we computed the KL divergence by using the phantom 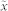 scaled with the effective photons emitted from each pixel/voxel. In the case of simulated data, we stopped the algorithms when they reached the minimum of the KL divergence (see Supplementary Information).

### Real images

To test the proposed algorithms we imaged the micro-tubule cytoskeleton of fixed PtK2 cells. We used two different protocols for labeling two different proteins of the filament systems. The first protocol localizes the *β*-tubulin protein; it involves a primary antibody (anti*β*-tubulin mouse IgG, Sigma) and a secondary antibody (sheep anti-mouse IgG, Dianova) labeled with ATTO 647N (Atto-Tec). The second protocol localizes keratin protein and uses the Citrine yellow fluorescent protein. The samples were examined using a Leica TCS STED-CW microscope (Leica Microsystems) equipped with a 100×/1.40 OIL STED orange objective (Leica Microsystems). The system is able to perform both confocal and STED imaging. We excited (with a regular Gaussian beam) ATTO 647N and Citrine fluorophores at 635 nm and 488 nm, respectively, and we collected emitted light in the 650–750 nm and 495–580 nm spectral windows, respectively. For STED imaging on Citrine tagged sample fluorescence we depleted with a doughnut shaped beam at 592 nm. In the case of real data, we stopped the algorithms when they reached convergence with a tolerance of 10^−3^ (Eq. (S26)).

### Computational features

Our test platform consists of a workstation equipped with 2 Intel Xeon Six-Core CPUs at 3.1 GHz, 188 GB of RAM and 4 GPUs Nvidia Fermi C2070. It is managed by a CentOS Linux distribution. Each GPU is highly parallel: 14 streaming multiprocessors for a total of 448 64 bit computing cores, a high-speed RAM block shared among the 448 cores and a cache. Additional hardware details are available in the last section of the Supplementary Information.

Two implementations of SGP are available: one in Matlab (CPU-based) and another one in C/CUDA (GPU-based). The GPU implementation is developed in mixed C and CUDA languages, the latter being a manufacturer-provided framework for C-like GPU programming (see Supplementary Information). We used Matlab v. 7.11 and CUDA v. 4.3.

The main computational cost in both the RL and the SGP iterations is a pair of forward and backward FFTs for computing the image convolutions.

In the GPU framework, these operations were faced by means of the Nvidia CUFFT library, while Matlab exploits a multi-threaded implemetation of FFTW libraries: a real-to-complex FFT is followed by a component-wise multiplication between the transformed iterate and the PSF and, at the end, a complex-to-real inverse FFT gives the convolution. A few additional component-wise operations are needed, which only depend on each single pixel/voxel (which is good for the GPU implementation), so that the complexity per iteration remains essentially unaffected. The SGP algorithm also requires a few products between long vectors, to update the steplengths: these are “reduction” operations, involving communications in the GPU case. They are performed by calling dedicated and optimized library routines (see Supplementary Information).

## Author Contributions

R.Z., G.Z., P.B., M.B. and G.V. designed the experiments. R.Z., G.Z., R.C., L.Z. and P.B. designed the algorithm and executed the experiments. R.Z., G.Z., L.Z., P.B., M.B. and G.V. analyzed the data. G.Z., L.Z., M.B. and G.V. wrote the manuscript. G.Z. and G.V. prepared the figures. All authors read and approved the final manuscript.

## Supplementary Material

Supplementary Information Supplementary Figures and Text

## Figures and Tables

**Table 1:** Scaled-gradient-projection (SGP) algorithm

*Initialization.*
Choose the starting point *x*^(0)^ ≥ 0, set the parameters *β*, *θ* ∈ (0, 1), 0 < α_min_ < α_max_, 0 < *L_1_* < *L_2_*.
For *k* = 0, 1, 2, … do the following steps:
1 Choose the parameter α*_k_* ∈ [α_min_, α_max_] and the scaling matrix 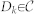 ;
2 Projection: 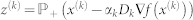 ; If z^(*k*)^ = *x*^(*k*)^ then stop: *x*^(*k*)^ is a minimum point; Endif
3 Descent direction: *d*^(*k*)^ = *z*^(*k*)^ − *x*^(*k*)^;
4 Set λ*_k_* = 1;
5 Backtracking loop:
If *f*(*x*^(*k*)^ + λ*_k_d*^(*k*)^) ≤ *f*(*x*^(*k*)^) + *βλ_k_*∇*f*(*x*^(*k*)^)*^T^d*^(*k*)^ Then
go to Step 6;
Else
set λ*_k_* = θλ*_k_* and go to Step 5;
Endif
6 Set *x*^(*k*+1)^ = *x*^(*k*)^ + λ*_k_d*^(*k*)^.
End
