# Supplementary material for: Towards real-time image deconvolution: application to confocal and STED microscopy
Source: Sci Rep. 2013 Aug 28;3:2523. doi: 10.1038/srep02523 (PMC3755287; doi:10.1038/srep02523)
Supplement: Supplementary Information — Supplementary Figures and Text [file srep02523-s1.pdf]

Supplementary Information

Towards real-time image deconvolution: application to confocal and STED microscopy

R. ZANELLA[1], G. ZANGHIRATI[2], R. CAVICCHIOLI[3], L. ZANNI[3], P. BOCCACCI[4], M. BERTERO[4] & G. VICIDOMINI[5,*]

[1]*Laboratorio delle Tecnologie per Terapie Avanzate, Università di Ferrara, Ferrara, Italy.*

[2]*Dipartimento di Matematica e Informatica, Università di Ferrara, Ferrara, Italy.*

[3]*Dipartimento di Scienze Fisiche, Informatiche e Matematiche, Università di Modena e Reggio Emilia, Modena, Italy.*

[4]*Dipartimento di Informatica, Bioingegneria, Robotica e Ingegneria dei Sistemi, Università di Genova, Genoa, Italy.*

[5]*Nanophysics, Istituto Italiano di Tecnologia, Genoa, Italy.*

[*] Correspondence should be addressed to G.V. (giuseppe.vicidomini@iit.it).

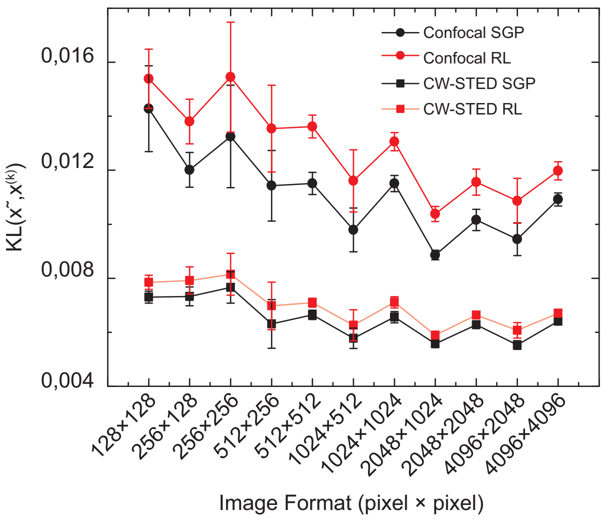

Figure 1: **Quality comparison between SGP and RL algorithms.** Kullback-Leibler divergence of the reconstructed image x_k from the known object $\tilde{x}$ as a function of the image format. Same conditions of Figures 1 and 2.

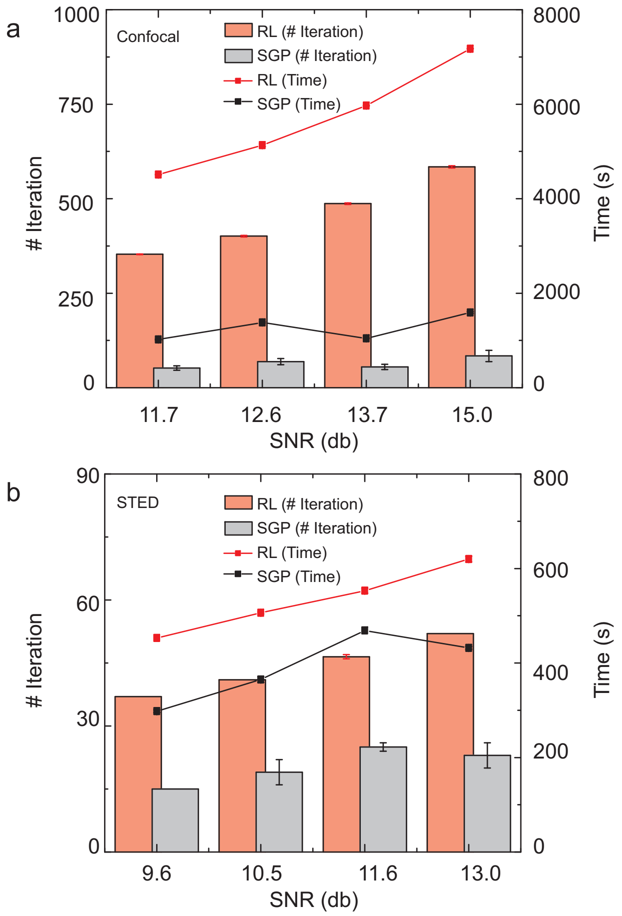

Figure 2: **Performance comparison between SGP and RL algorithms as function of SNR.** (a,b) Number of iteration and computational time as a function of the the SNR for CLSM (a) and STED microscopy (b). Same conditions of Figures 1 and 2. Image format 4096×4096. The SNR is controlled by changing the factor $\tau = 2, 3, 5, 10$ (Eq. (10)), respectively.

Richardson-Lucy (RL) algorithm

Thanks to the PSF normalization of equation 1 (Main Text) and the modification introduced to account for background contribution [1], the RL algorithm takes the following form:

– given $x^{(0)}$, for $k = 0, 1, 2, ...$ compute

$$x^{(k+1)} = x^{(k)} \cdot H^T \left(\frac{y}{Hx^{(k)} + b} \right) \quad . \tag{1}$$

Here, and in the following, the multiplication of two arrays/cubes indicates the component-wise product; similarly the fraction of two arrays/cubes indicates component-wise quotient.

If we remark that the gradient of the KL-divergence is given by

$$\nabla_x f_0(x; y) = \hat{1} - H^T \left(\frac{y}{Hx + b} \right) \quad , \tag{2}$$

where $\hat{1}$ indicates equation 1 can be written as follows

$$x^{(k+1)} = x^{(k)} - x^{(k)} \cdot \nabla_x f_0(x^{(k)}; y) \tag{3}$$

and therefore it is a scaled gradient method with scaling $x^{(k)}$ at iteration k.

Split-Gradient method (SGM)

As concerns SGM for the minimization of the function defined by equations 3 and 2 (Main Text), if $U_1(x), V_1(x)$ is a pair of nonnegative arrays/cubes such that

$$-\nabla_x f_1(x) = U_1(x) - V_1(x) \quad , \tag{4}$$

then the algorithm is as follows:

– given $x^{(0)}$, for $k = 0, 1, 2, ...$ compute

$$x^{(k+1)} = \frac{x^{(k)}}{\hat{1} + \beta V_1(x^{(k)})} \cdot \left\{ H^T \left(\frac{y}{Hx^{(k)} + b} \right) + \beta \; U_1(x^{(k)}) \right\} \quad . \tag{5}$$

It is easy to see that this iteration can be written as a scaled gradient step:

$$x^{(k+1)} = x^{(k)} - \frac{x^{(k)}}{\hat{1} + \beta V_1(x^{(k)})} \cdot \nabla_x f_\beta(x^{(k)}; y) \quad . \tag{6}$$

Convergence of SGM can be obtained by a standard line-search strategy that ensures both the descent of the objective function $f_\beta(x;y)$ and the nonnegativity of $x^{(k+1)}$. Expressions of the functions $U_1(x), V_1(x)$ for a number of regularization functions are given, for instance, in [2, 3, 4].

For the convenience of the reader we provide a very simple argument for justifying SGM. From the stationarity condition provided by the second Karush-Kuhn-Tucker (KKT) condition in the case of nonnegativity constraint, using the decomposition of the gradient of $f_1(x)$ given in equation 4, we obtain for a stationary point x^* the condition

$$\begin{aligned} 0 &= x^* \cdot \nabla_x f_\beta(x^*;y) \\ &= x^* - x^* \cdot H^T\left(\frac{y}{Hx^*+b}\right) + \beta x^* \cdot V_1(x^*) - \beta x^* \cdot U_1(x^*) \quad , \end{aligned} \tag{7}$$

which can be written as the following fixed point equation

$$x^* = \frac{x^*}{\hat{1} + \beta V_1(x^*)} \cdot \left\{ H^T\left(\frac{y}{Hx^*+b}\right) + \beta U_1(x^*) \right\} \quad . \tag{8}$$

By applying the method of successive approximations we obtain the iterative algorithm of equation 5.

In the case of quadratic (or Tikhonov) regularization, *i.e.*,

$$f_1(x) = \frac{1}{2}\sum_{i\in S} x_i^2 \quad , \tag{9}$$

we have $\nabla_x f_1(x) = x \geq 0$ and the splitting $V_1(x) = x,\ U_1(x) = 0$ can be used in SGM. Then, using again the stationarity condition, we obtain for a stationary point x^*

$$0 = x^* \cdot \nabla_x f_\beta(x^*;y) = x^* - x^* \cdot H^T\left(\frac{y}{Hx^*+b}\right) + \beta(x^*)^2 \quad , \tag{10}$$

or also

$$x^* + \beta(x^*)^2 \quad = x^* \cdot H^T\left(\frac{y}{Hx^*+b}\right) \quad . \tag{11}$$

If we write this relationship as the following fixed point equation

$$x^* = \frac{x^*}{\hat{1} + \beta x^*} \cdot H^T\left(\frac{y}{Hx^*+b}\right) \quad , \tag{12}$$

by applying the method of successive approximation we obtain the SGM iteration for quadratic regularization:

– given $x^{(0)}$, for $k = 0, 1, 2, ...$ compute

$$x^{(k+1)} = \frac{x^{(k)}}{\hat{1} + \beta x^{(k)}} \cdot H^T\left(\frac{y}{Hx^{(k)}+b}\right) \quad . \tag{13}$$

Conchello-McNally method

The previous algorithm can be considered as an alternative to the expectation-maximization (EM) algorithm proposed by Conchello-McNally [5] which is frequently used in the deconvolution of microscopy images:

- given $x^{(0)}$, for $k = 0, 1, 2, ...$ compute

$$\begin{aligned} u^{(k+1)} &= x^{(k)} \cdot H^T \left(\frac{y}{Hx^{(k)} + b} \right) \ , \\ x^{(k+1)} &= -\frac{1}{2\beta} \left(\hat{1} - \sqrt{\hat{1} + 4\beta u^{(k+1)}} \right) . \end{aligned} \tag{14}$$

Since SGM and Conchello-McNally algorithms are simple modification of the RL algorithm we expect that their convergence is slow. However SGM can be accelerated by inserting its scaling in SGP.

It is interesting to show that this method can also be considered as an iterative method for solving the fixed point equation given in equation 11. Indeed, if we write this equation as follows

$$u^* = x^* \cdot H^T \left(\frac{y}{Hx^* + b} \right) \ \ , \ \ x^* + \beta (x^*)^2 \ \ = u^* \ \ , \tag{15}$$

and we consider the second equation as a second degree algebraic equation for given u^*, we obtain

$$u^* = x^* \cdot H^T \left(\frac{y}{Hx^* + b} \right) \ \ , \ \ x^* = -\frac{1}{2\beta} \left(\hat{1} - \sqrt{\hat{1} + 4\beta u^*} \right) \ \ . \tag{16}$$

Therefore the EM method of [5], given the iteration $x^{(k)}$, consists in updating first u by means of the first relation and then to update x by means of the second relation with u^* replaced by $u^{(k+1)}$.

We conclude by remarking that x^*, as given by the second relation in equation 16, is the solution of the following denoising problem

$$x^* = \text{argmin} \sum_{i \in S} \left\{ u_i^* \ln \frac{u_i^*}{x_i} + x_i - u_i^* + \frac{1}{2} \beta (x_i)^2 \right\} \ \ . \tag{17}$$

Therefore the Conchello-McNally algorithm can also be viewed as a RL iteration followed by a denoising of the iterate, assuming Poisson noise and quadratic regularization.

Boundary effect correction

If the unknown object x extends up to the boundary of the image domain so that the effect of the PSF is to generate an image which is not completely contained in the field-of-view of the microscope, then the previous deconvolution methods produce annoying boundary artifacts. Several methods have been proposed for removing this effect but in this paper we focus on an approach proposed in [6] for application to the RL algorithm; it can also be easily extended to the case of regularized deconvolution. Here we give the equations in such a case showing the application to SGP. The non-regularized algorithms can be obtained by setting $\beta = 0$.

The idea is to reconstruct the object over a domain broader than that of the detected image and to merge, by zero padding, the arrays/cubes of the image and of the object into arrays/cubes with a dimension such that their Fourier transforms can be computed by means of FFT. We denote by $\bar{S}$ the set of values of the index labeling the pixels/voxels of the broader array/cube, by R that of the object array/cube and by S that of the image array/cube, so that $S \subset R \subset \bar{S}$. It is obvious that also the PSF must be defined over $\bar{S}$ and this can be done in different ways, depending on the specific problem one is considering. In microscopy the extension can be in general performed by zero padding. We point out that the PSF must be normalized to unit volume over $\bar{S}$. The reconstruction of the object outside S is not reliable in most cases but its reconstruction inside S is practically free of boundary artifacts, as shown in [6].

If we denote by M_R, M_S the arrays/cubes, defined over $\bar{S}$, which are 1 over R, S respectively and 0 elsewhere, we introduce the following matrices H and H^T:

$$(Hu)_i = (M_S)_i \sum_{j \in \bar{S}} h_{i-j} (M_R)_j u_j \tag{18}$$

$$(H^T v)_j = (M_R)_j \sum_{i \in \bar{S}} h_{i-j} (M_S)_i v_i \quad , \tag{19}$$

where u and v denote generic arrays/cubes defined over $\bar{S}$. Both matrices can be easily computed by means of FFT. Then, with these definitions, the data fidelity function is given again by equation 2 (Main Text), with S replaced by $\bar{S}$, so that its gradient is now given by

$$\nabla_x f_0(x; y) = \alpha - H^T \left(\frac{y}{Hx + b} \right) \quad , \tag{20}$$

where α is the array/cube defined by

$$\alpha_j = (H^T \hat{1})_j = (M_R)_j \sum_{i \in \bar{S}} h_{i-j} (M_S)_i \quad . \tag{21}$$

We remark that the quantity multiplying M_R in this equation can be used for defining the reconstruction domain R. Indeed, thanks to the normalization of the PSF, each value α_j is ≤ 1 and is a measure of the fraction of the pixel/voxel j contributing to the image; it can be very small or zero for pixels/voxels of $\bar{S}$, depending on the behaviour of the PSFs. Therefore, given a thresholding value σ (for instance $\sigma = 10^{-2}$), we use the following definition

$$R = \left\{ j \in \bar{S} \,\middle|\, \sum_{i \in \bar{S}} h_{i-j} (M_S)_i \geq \sigma \right\} \quad . \tag{22}$$

Then the SGM algorithm, with boundary effect correction, is given by

$$x^{(k+1)} = M_R \cdot \frac{x^{(k)}}{\alpha + \beta\, V_1(x^{(k)})} \cdot \left\{ H^T \left(\frac{y}{Hx^{(k)} + b} \right) + \beta\, U_1(x^{(k)}) \right\} \quad , \tag{23}$$

so that each iteration is zero in the pixels/voxels outside R. It is easy to show that also this algorithm is a scaled gradient method.

As concerns SGP, the boundary effect correction is introduced by modifying the scaling matrix as follows

$$D_k = \operatorname{diag} \left(M_R \cdot \min \left[L_2, \max \left\{ L_1, \frac{x^{(k)}}{\alpha + \beta V_1(x^{(k)})} \right\} \right] \right) \tag{24}$$

while all the other steps remain unchanged.

SGP implementation

Initialization

As far as the initial point $x^{(0)}$ concerns, a nonnegative image is required. The possible choices implemented in our code are:

- a constant image with pixel values equal to the background-subtracted flux of the noisy data divided by the number of pixels/voxels;

- any input image provided by the user.

For ensuring a feasible initial point, the given $x^{(0)}$ is projected on the feasible region before to start the iterative minimization procedure. The constant image has been chosen for our numerical experiments; in the case of boundary effect correction, only the pixels/voxels in R become equal to a constant value, while the remaining values of $\bar{S}$ are set to zero. The constant value is normalized in such a way that the flux of the constant image over R coincides with the flux of the background-subtracted image over S.

Step-length selection

In the case of non-scaled gradient method (D_k equal to the identity matrix) it is possible to prove that, if ${s^{(k-1)}}^T w^{(k-1)} > 0$, then the inequality $0 < \alpha_k^{(BB2)} \leq \alpha_k^{(BB1)}$ holds for the BB step-lengths. For unconstrained quadratic minimization problems this property is exploited for designing effective alternation strategies of the two BB rules, able to improve significantly the convergence rate of the gradient schemes. In particular, the alternation strategies are useful for generating step-lengths that better approximate the inverse of the eigenvalues of the objective Hessian, that is a crucial property in accelerating gradient methods [7, 8]. Numerical evidence is available that confirms the efficiency of these alternated BB rules also in case of non-linear constrained minimization problems [9, 10, 11, 12].

When scaled gradient directions are exploited, the inequality $\alpha_k^{(BB2)} \leq \alpha_k^{(BB1)}$ can not be proved for the step-lengths of equation 5; nevertheless, in our experiments this inequality has been often observed and the alternation strategies proposed for the non-scaled methods still provide interesting convergence rate improvements [13, 14, 15]. For this reason, we have equipped our SGP version with the step-lengths alternation criterion proposed in [8], that is one of the most effective rule for non-scaled gradient methods. We provide a description of this criterion in the following.

First of all the values produced by equation 5 (Main Text) are constrained into the interval $[\alpha_{min}, \alpha_{max}]$:

IF ${s^{(k-1)}}^T D_k^{-1} w^{(k-1)} \leq 0$ THEN
$\alpha_k^{(1)} = \min\{10 \cdot \alpha_{k-1},\ \alpha_{max}\}$;
ELSE
$\alpha_k^{(1)} = \min\left\{\alpha_{max},\ \max\left\{\alpha_{min},\ \alpha_k^{(BB1)}\right\}\right\}$;
ENDIF
IF ${s^{(k-1)}}^T D_k w^{(k-1)} \leq 0$ THEN
$\alpha_k^{(2)} = \min\{10 \cdot \alpha_{k-1},\ \alpha_{max}\}$;
ELSE

$\alpha_k^{(2)} = \min \left\{ \alpha_{max}, \ \max \left\{ \alpha_{min}, \ \alpha_k^{(BB2)} \right\} \right\};$
ENDIF

Then the following adaptive alternation is performed:

IF $\alpha_k^{(2)}/\alpha_k^{(1)} \leq \tau_k$ THEN

$$\alpha_k = \min_{j=\max\{1,k+1-M_\alpha\},\ldots,k} \{ \alpha_j^{(2)} \} \tag{25}$$

$\tau_{k+1} = 0.9 \cdot \tau_k;$
ELSE
$\alpha_k = \alpha_k^{(1)}; \qquad \tau_{k+1} = 1.1 \cdot \tau_k;$
ENDIF

where M_α is a prefixed positive integer and $\tau_1 \in (0, 1)$. In contrast to the criterion proposed in [8], here a variable threshold τ_k is exploited with the aim of avoiding the selection of the same rule for a too large number of iterations. Furthermore, in our experience, the use of the BB values provided by equation 25 (that are generally lower than those provided by $\alpha_k^{(1)}$) in the first iterations slightly improves the reconstruction accuracy and, consequently, in the proposed SGP version we start the step-length alternation only after the first 20 iterations.

Parameters setting

Even if the number of SGP parameters is certainly higher than that of the RL and SGM approaches, the huge amount of tests carried out in several applications led to an optimization of these values which allows the user to have at his disposal a robust approach without the need of a problem-dependent parameters tuning. Some of these values have been fixed according to the original paper [13], as the line-search parameters β and θ which have been set equal to 10^{-4} and 0.4, respectively. Also most of the step-length parameters remained unchanged, as $\alpha_0 = 1.3$, $\tau_1 = 0.5$, $\alpha_{max} = 10^5$ and $M_\alpha = 3$, while α_{min} has been set equal to 10^{-5}.

The main change with respect to [13] concerns the choice of the bounds (L_1, L_2) for the scaling matrices. While in the original paper the choice was a couple of fixed values $(10^{-10}, 10^{10})$, independent of the data, we decided to automatically adapt these bounds to the input image: we perform one step of the RL method and tune the parameters (L_1, L_2) according to the min/max values of the resulting image w according to the rule

IF $w_{\max}/w_{\min} < 50$ THEN
 $L_1 = w_{\min}/10$;
 $L_2 = w_{\max} \cdot 10$;
ELSE
 $L_1 = w_{\min}$;
 $L_2 = w_{\max}$;
ENDIF

Stopping rules

As mentioned in the main text, in the case of ML estimates both RL and SGP must not be pushed to convergence and an early stopping of the iterations is required to achieve reasonable reconstructions. In our code, we introduced different stopping criteria, which can be adapted by the user according to his/her purposes:

- fixed number of iterations. The user can decide how many iterations of SGP must be done.
- convergence of the algorithm. In such a case, a stopping criterion based on the convergence of the objective function to its minimum value is introduced. Iteration is stopped when

  (26) $$|f_\beta(x^{(k+1)}; y) - f_\beta(x^{(k)}; y)| \le \texttt{tol}\ f_\beta(x^{(k+1)}; y) \quad ,$$

  where `tol` is a parameter that can be selected by the user.
- minimization of the reconstruction error. This criterion can be used in a simulation study. If one knows the object $\widetilde{x}$ used to generate the synthetic image, then one can stop the iterations when the relative reconstruction error

  (27) $$\rho^{(k)} = \frac{|x^{(k)} - \widetilde{x}|}{|\widetilde{x}|}$$

  reaches a minimum value. A very frequently used measure of error is given by the ℓ_2 norm, *i.e.*, $|\cdot| = \|\cdot\|_2$ and this is the criterion implemented in our code.
- minimization of the Kullback-Leibler divergence of the reconstructed image $x^{(k)}$ from the known object $\tilde{x}$

  (28) $$\mathrm{KL}(\tilde{x}, x^{(k)}) = \frac{1}{\#S} \sum_{i \in S} \left[\tilde{x}_i \ln\left(\frac{\tilde{x}_i}{x_i^{(k)}} \right) + \left(x_i^{(k)} - \tilde{x}_i \right) \right] \quad .$$

  Also this case can be applied only on simulation study.

- the use of a discrepancy criterion. In the case of real data, one can use a given value of some measure of the "distance" between the real data and the data computed by means of the current iteration. A recently proposed criterion consists in defining the "discrepancy function" for an image y of size $N \times N$ as follows [16]

  $$\mathcal{D}^{(k)} = \frac{2}{N^2} f_0(x^{(k)}; y) \quad , \tag{29}$$

  and stopping the iterations when $\mathcal{D}^{(k)} = \eta$, where η is a given number close to 1. Work is in progress to validate this discrepancy criterion with the purpose of obtaining rules of thumb for estimating η in real applications.

The last stopping rule deserves a few comments. In [16] it is shown that, if $\widetilde{x}$ is the object generating the noisy image y, then the expected value of $f_0(\widetilde{x}; y)$ is close to $N^2/2$. This property is used to select a value of the regularization parameter when the image reconstruction problem is formulated as the minimization of the KL divergence with the addition of a suitable regularization term. This use is justified by evidence that in some important cases it provides a unique value of the regularization parameter. Moreover, it has also been shown that the quantity $\mathcal{D}^{(k)}$, defined in equation (29), decreases for increasing k, starting from a value greater than 1. Therefore, it can be used as a stopping criterion. Preliminary numerical experiments described in that paper show that it can provide a sensible stopping rule at least in simulation studies.

Testing platform

This appendix gives some deeper details on our testing platform. As we mentioned, each one of the 4 GPUs in the workstation has 14 streaming multiprocessors. All these streaming multiprocessors can access the system's main global memory where, typically, the problem data are stored. The connection bus with the cores has a bandwidth of 144 GB/sec. The whole system's peak computing performance is over 2.5 Tflops for double precision arithmetic. Each GPU board is connected to the CPU with a 16x PCI-Express bus, which grants a 16 GB/sec transfer rate. It should be noticed that the CPU-to-GPU transfer speed is much slower than the GPU-to-GPU transfer: hence, to maximize the GPU benefits, it is very important to reduce the CPU-to-GPU memory communications and keep all the problem data into the GPU memory.

Nvidia provides a programming framework called CUDA (Compute Unified Device Architecture) for its GPUs. For further information see `http://www.nvidia.com/cuda`. By means of CUDA, it is possible to program a GPU using a C-like programming language. In this paradigm the CPU controls the instruction flow, the communications with the peripherals and starts the single computing tasks on the GPU. The GPU, composed by a number of streaming multiprocessors, performs the raw computation tasks, using the problem data stored into the graphics memory. In our numerical experiments we used CUDA 4.3.

The full GPU-to-GPU bandwidth can be obtained only if a coalesced memory access scheme is used (see the Nvidia documentation [17]). This is the reason why our GPU computational kernels are implemented using that memory pattern. Two CUDA kernel libraries are very important in our implementation of the SGP algorithm: CUFFT and CUBLAS. The former enables 1D, 2D and 3D FFTs (complex-to-complex, real-to-complex and complex-to-real versions are all available). The latter provides GPU implementations of the well known level 1, 2 and 3 BLAS libraries. These libraries are strongly recommended for maximizing GPU performances.

In the C++ implementation, the FFT is provided by the well known FFTW library [18, 19], a C library for computing the discrete Fourier transform (DFT) of multidimensional real or complex data of arbitrary size.

The pixel/voxel additional component-wise operations are localized, that is, the result in each pixel/voxel does not depend on that of any other: hence, those opoerations can be easily distributed on all the available processors, which makes them particularly suited for the GPU implementation. It must be noticed that the scalar products for updating the step-length α_k from (5) is a performance-critical part of the SGP algorithm, because they require a large number of inter-processor communications and values dependencies prevent a straight parallelization. To face the bottleneck, we used the `reduce` function provided by the Thrust library [20], which generally achieves remarkable performances while retaining sufficient stability.

Upcoming next-generation GPU architectures, such as Nvidia's Maxwell series, could further improve the CUDA computational performances for the largest-scale images, thanks to the improved CPU-GPU connection and the increased double-precision peak performance. However, for the case of very large 3D reconstructions, the overall amount of memory local to each board will likely remain the limiting factor.

References

[1] Snyder, D. L. & Hammoud, A. M. Image recovery from data acquired by a charge-coupled-device camera. *J. Opt. Soc. America A* **10**, 1014–1023 (1993).

[2] Lantéri, H., Roche, M. & Aime, C. Penalized maximum likelihood image restoration with positivity constraints: multiplicative algorithms. *Inverse Problems* **18**, 1397–1419 (2002).

[3] Vicidomini, G., Boccacci, P., Diaspro, A. & Bertero, M. Application of the split-gradient method to 3d image deconvolution in fluorescence microscopy. *J. Microsc.* **234**, 47–61 (2009).

[4] Staglianò, A., Boccacci, P. & Bertero, M. Analysis of an approximate model for poisson data reconstruction and a related discrepancy principle. *Inverse Problems* **27**, 125003 (2011).

[5] Conchello, J. A. & McNally, J. G. Fast regularization technique for expectation maximization algorithm for optical sectioning microscopy. *Proc. SPIE* **2655**, 199–208 (1996).

[6] Bertero, M. & Boccacci, P. A simple method for the reduction of boundary effects in the richardson-lucy approach to image deconvolution. *Atron. Astrophys.* **437**, 369–374 (2005).

[7] Zhou, B., Gao, L. & Dai, Y. Gradient methods with adaptive step-sizes. *Comput. Optim. Appl.* **35**, 69–86 (2006).

[8] Frassoldati, G., Zanghirati, G. & Zanni, L. New adaptive stepsize selections in gradient methods. *J. Indutrial and Management Optimization* **4**, 299–312 (2008).

[9] Serafini, T., Zanghirati, G. & Zanni, L. Gradient projection methods for quadratic programs and applications in training support vector machines. *Opt. Methods and Software* **20**, 353–378 (2005).

[10] Dai, Y. & Fletcher, R. Projected barzilai-borwein methods for large-scale box-constrained quadratic programming. *Numerische Mathematik* **100**, 21–47 (2005).

[11] Zanni, L. An improved gradient projection-based decomposition technique for support vector machines. *Computational Management Science* **3**, 131–145 (2006).

[12] Loris, I., Bertero, M., Mol, C. D., Zanella, R. & L.Zanni. Accelerating gradient projection methods for ℓ_1-constrained signal recovery by steplength selection rules. *Appl. Comput. Harmon. A.* **27**, 247–254 (2009).

[13] Bonettini, S., Zanella, R. & Zanni, L. A scaled gradient projection method for constrained image deblurring. *Inverse Problems* **25**, 015002 (23pp) (2009).

[14] Favati, P., Lotti, G., Menchi, O. & Romani, F. Performance analysis of maximum likelihood methods for regularization problems with nonnegativity constraints. *Inverse Problems* **26**, 085013 (18pp) (2010).

[15] Zanella, R., Boccacci, P., Zanni, L. & Bertero, M. Efficient gradient projection methods for edge-preserving removal of Poisson noise. *Inverse Problems* **25**, 045010 (2009).

[16] Bertero, M., Boccacci, P., Talenti, G., Zanella, R. & Zanni, L. A discrepancy principle for poisson data. *Inverse Problems* **26**, 105004 (2010).

[17] NVidia Corp. *NVIDIA CUDA – Compute Unified Device Architecture. Programming Guide* (2011).

[18] Frigo, M. & Johnson, S. G. *FFTW – Fastest Fourier Transform in the West* (2011). URL `http://www.fftw.org/`.

[19] Frigo, M. & Johnson, S. G. The design and implementation of FFTW3. *Proceedings of the IEEE* **93**, 216–231 (2005). Special issue on “Program Generation, Optimization, and Platform Adaptation”.

[20] Hoberock, J. & Bell, N. Thrust: A parallel template library (2010). URL `http://www.meganewtons.com/`. Version 1.3.0.
